# Connexin 43 Hemichannels Regulate Osteoblast to Osteocyte Differentiation

**DOI:** 10.3389/fcell.2022.892229

**Published:** 2022-05-27

**Authors:** Rui Hua, Sumin Gu, Jean X. Jiang

**Affiliations:** Department of Biochemistry and Structural Biology, University of Texas Health Science Center, San Antonio, TX, United States

**Keywords:** IDG-SW3 cells, Cx43, CRISPR/Cas9, osteoblast differentiation, mineralization, osteoclastogenesis

## Abstract

Connexin 43 (Cx43) is the predominant connexin subtype expressed in osteocytes. Osteocytes, accounting for 90%–95% of total bone cells, function as orchestrators coordinating balanced activity between bone-resorbing osteoclasts and bone-forming osteoblasts. In this study, two newly developed osteocytic cell lines, OCY454 and IDG-SW3, were used to determine the role of Cx43 gap junctions and hemichannels (HCs) in the regulation of osteoblast to osteocyte differentiation. We found that the Cx43 level was substantially increased during the differentiation of IDG-SW3 cells and is also much higher than that of OCY454 cells. We knocked down Cx43 expression using the lentiviral CRISPR/Cas9 approach and inhibition of Cx43 HCs using Cx43 (E2) antibody in IDG-SW3 cells. Cx43 knockdown (KD) or Cx43 HC inhibition decreased gene expression for osteoblast and osteocyte markers, including alkaline phosphatase, type I collagen, dentin matrix protein 1, sclerostin, and fibroblast growth factor 23, whereas increasing the osteoclastogenesis indicator and the receptor activator of nuclear factor kappa-B ligand (RANKL)/osteoprotegerin (OPG) ratio at early and late differentiation stages. Moreover, mineralization was remarkably attenuated in differentiated Cx43-deficient IDG-SW3 cells compared to ROSA26 control. The conditioned medium collected from fully differentiated IDG-SW3 cells with Cx43 KD promoted osteoclastogenesis of RAW264.7 osteoclast precursors. Our results demonstrated that Cx43 HCs play critical roles in osteoblast to osteocyte differentiation process and regulate osteoclast differentiation *via* secreted factors.

## Introduction

Osteocytes are the most abundant cell type in bone tissue, comprising 90–95% of bone cells. They are buried within the mineral bone matrix and form an extensive network through long dendritic processes, which allows osteocytes to communicate with neighboring osteocytes, bone-forming osteoblasts, and bone-resorbing osteoclasts (Creecy et al., 2020; Robling and Bonewald, 2020). Emerging studies suggest that osteocytes function as master orchestrators of bone remodeling (Dallas et al., 2013; Schaffler et al., 2014). Osteocytes are shown to sense mechanical loading to coordinate adaptive responses of the skeleton (Qin et al., 2020) and actively secrete factors that regulates phosphate homeostasis and mineral metabolism (Chande and Bergwitz, 2018).

Osteocytes are derived from osteoblasts through osteogenesis. During this transition process, osteoblasts lay down osteoid (non-mineralized bone matrix), accompanied by a morphology change from polygonal to highly dendritic with reduced cell volume, and eventually transform into osteocytes embedded in the mineralized bone matrix (Franz-Odendaal et al., 2006; Dallas and Bonewald, 2010). Since osteocytes reside within the mineralized lacuno-canalicular network, their isolation has been difficult, generating low yields and high heterogeneity. In addition, osteocytes, as terminally differentiated cells, tend to lose phenotype when isolated from their natural mineralized environment (Dallas et al., 2013; Wang et al., 2019). Despite the abundance and importance, osteocytes have been a challenge to study due to lack of appropriate *in vitro* cell models. It is not until the past 2 decades or so that significant osteocyte cell models have been developed. MLO-Y4 is the first established osteocyte-like cell line, which has been one of the most widely used for studying osteocyte functions (Kato et al., 1997). This cell line was derived from the long bones of transgenic mice expressing the immortalizing SV40 T antigen driven by the osteocalcin promoter. However, several limitations of MLO-Y4 cells include the absence of mineralized matrix, constitutive expression of the large T antigen, and very low levels of the mature osteocyte markers fibroblast growth factor 23 (FGF23) and sclerostin (SOST) (Yang et al., 2009; Woo et al., 2011).

There are two pre-osteocyte cell models, IDG-SW3 (Woo et al., 2011) and OCY454 (Spatz et al., 2015). These two cell lines were generated by crossing the dentin matrix protein 1 (DMP1)–GFP transgenic mice (Kalajzic et al., 2004) with the “immortomouse”, which carries a temperature-sensitive SV40 T antigen (Jat et al., 1991). When cultured at 33°C, both IDG-SW3 and OCY454 cells proliferate rapidly. However, at 37°C, they no longer express the SV40 T antigen and differentiate from the late osteoblast to the late osteocyte, closely recapitulating the phenotype of primary cells. These two cell lines provide valuable tools for studying the transition from osteoblast to mature osteocyte. The expression of FGF23 mRNA is elevated in response to 1,25-dihydroxyvitamin D_3_ treatment, while the SOST expression with parathyroid hormone (PTH) treatment is downregulated in IDG-SW3 cells (Woo et al., 2011). OCY454 cells express SOST at earlier time points than IDG-SW3 cells after the induction of differentiation, and the expression is upregulated in response to microgravity *in vitro* (Spatz et al., 2015).

Connexin 43 (Cx43) is the most abundant connexin present in osteocytes, acting as a key modulator for skeletal homeostasis (Batra et al., 2012; Stains and Civitelli, 2016; Hua et al., 2021). Cx43 forms gap junctions, which mediate direct cell–cell communication. Cx43 hemichannels (HCs) are unpaired gap junction channels, mediating communication between cells and their extracellular environment. Connexin-formed gap junctions and HCs allow the passage of small molecules (MW < 1 kDa) such as ions, essential metabolites, and secondary messengers, including Ca^2+^, NAD^+^, prostaglandin E_2_ (PGE_2_), cAMP, ADP, and ATP (Goodenough et al., 1996). Deficiency of Cx43 causes heart deficits and death of animals soon after birth (Reaume et al., 1995; Ya et al., 1998), accompanied by osteoblast dysfunction and delayed intramembranous and endochondral ossification in fetuses or newborn pups (Lecanda et al., 2000; Chaible et al., 2011). Using osteoblast- and osteocyte-specific Cx43 knockout mouse models, it has been reported that Cx43 contributes to bone cell proliferation, survival, and differentiation (Plotkin et al., 2008; Watkins et al., 2011; Bivi et al., 2012). Our previous work showed that impairment of Cx43 HCs in osteocytes negatively affect bone formation, remodeling, and osteocyte viability (Xu et al., 2015).

In this study, we aim to investigate the role of Cx43 in regulating osteoblast to osteocyte differentiation, and its impact on osteoclastogenesis. Taking advantage of the newly developed *in vitro* osteocytic cell models, we established a Cx43 knockdown (KD) stable cell line using lentiviral-mediated CRISPR/Cas9 genome editing technology and specifically inhibited Cx43 HCs using Cx43 (E2) antibody. We evaluated osteoblastic and osteocytic marker genes expression and mineralization at different differentiation stages as well as the regulation on osteoclastogenesis. This study will help gain new insights into the basic regulatory mechanisms of osteocyte differentiation and implications for the pathogenesis and treatment of osteoporosis.

## Materials and Methods

### Cell Culture

IDG-SW3 cells, a gift from Dr. Lynda Bonewald (Indiana University), were cultured on collagen-coated (rat tail collagen type I, Corning, 354236, 0.15 mg/ml) plates (Woo et al., 2011). Cells were expanded in immortalizing conditions in an α-MEM medium (Thermo Fisher Scientific, Waltham, MA, United States) supplemented with 10% fetal bovine serum (FBS), 50 U/mL of IFN-γ (Sigma-Aldrich, IF005, St. Louis, MO, United States), and 1% penicillin/streptomycin at 33°C and 5% CO_2_. For osteogenesis induction, cells were cultured in the α-MEM medium supplemented with 10% FBS, 50 μg/ml ascorbic acid, and 4 mM β-glycerophosphate at 37°C and 5% CO_2_. For Cx43 (E2) antibody treatment, IDG-SW3 cells were supplied with 2 μg/ml Cx43 (E2) antibody upon differentiation, the medium was changed every 2 days.

OCY454 cells were kindly provided by Dr. Paola Divieti Pajevic (Boston University). Cells were expanded in the α-MEM medium supplemented with 10% FBS and 1% penicillin/streptomycin at 33°C and 5% CO_2_ on collagen-coated plates, as described previously (Spatz et al., 2015). Upon confluence, cells were plated on non-collagen–coated plates to induce osteogenesis in a 5% CO_2_ incubator at 37°C.

### Plasmid Design and Construction

The 20-nucleotide single guide RNA (sgRNA) sequences were designed using the CRISPR design and optimization tool (CRISPR-DO) (Ma et al., 2016). SgRNA oligos were annealed and cloned into the BsmBI enzyme site of the lentiviral expression vector lentiCRISPRv2 (Addgene, plasmid #52961, Watertown, MA, United States) (Sanjana et al., 2014; Shalem et al., 2014). The sgRNA sequences used in this study are as follows: Cx43 KD-1: 5′-AAG​CCT​ACT​CCA​CGG​CCG​G-3′; Cx43 KD-2: 5′- AAA​GTG​GCG​CAG​ACC​GAC​G-3′; and ROSA26: 5′- CAC​CGC​GCC​CAT​CTT​CTA​GAA​AGA​C-3′.

### Lentivirus Packaging and Infection

The HEK293T cells (ATCC, CRL-11268, Manassas, VA, United States) were grown at 37°C and 5% CO_2_ in Dulbecco’s modified Eagle’s medium (Thermo Fisher Scientific), and lentiviruses were produced by transfecting the HEK293T cells with lentiCRISPRv2:Cx43-sgRNA or ROSA26-sgRNA, together with helper plasmids pCMV-VSV-G (Addgene, plasmid #8454) and psPAX2 (Addgene, plasmid #12260). The transfections were carried out using Lipofectamine 2000 (Thermo Fisher Scientific, 11668019), according to manufacturer’s instructions. The virus-containing medium was harvested 48 and 72 h after transfection and subsequently pre-cleaned with a 0.45 μm filter (Millipore, Burlington, MA, United States), as previously described (Jiang et al., 2015; Wang et al., 2016). The viral supernatant was added to IDG-SW3 cells with polybrene. The media was changed 24 h after the infection. An antibiotic kill curve experiment was performed to determine the optimal concentration of puromycin needed to eliminate untransduced cells. At 48 h postinfection, cells were selected with 5 μg/ml puromycin (Sigma-Aldrich, P8833) for 5 days.

### Preparation of Cell Membrane Extracts and Western Blotting

Cultured cells were collected in lysis buffer (5 mM Tris, 5 mM EDTA/EGTA, and proteinase inhibitors) and then ruptured by pipetting using a 20-gauge needle. Cell lysates were first centrifuged at 1,000 rpm for 5 min. The supernatant was transferred into ultracentrifuge tubes (Beckman Coulter, 357448, Brea, CA, United States) and centrifuged at 45,000 g for 45 min. The pellet was resuspended in lysis buffer, and the membrane protein was dissolved by addition of SDS to a 1% final concentration. Protein concentrations of SDS-dissolved lysates were determined by using a Micro BCA Protein Kit (Thermo Scientific, 23235), and the lysates were used for Western blotting analysis. Each protein sample was boiled in SDS loading buffer, subjected to electrophoresis on a 10% SDS-polyacrylamide gel, and electroblotted on a nitrocellulose membrane. Membranes were incubated with affinity-purified polyclonal Cx43 antibody (1:300) (Cherian et al., 2003), polyclonal GFP antibody (Abcam, ab290, 1:2000 dilution, Cambridge, United Kingdom), monoclonal β-actin antibody (Thermo Fisher Scientific, MA515739, 1:5000 dilution), or monoclonal GAPDH antibody (Thermo Fisher Scientific, AM4300, 1:5000 dilution). Primary antibodies were detected with goat anti-rabbit IgG–conjugated IRDye^®^ 800CW and goat anti-mouse IgG–conjugated IRDye^®^ 680RD (1:15000 dilution) using a LiCor Odyssey Infrared Imager (LI-COR, Lincoln, NE, United States), as previously described (Ma et al., 2019). The band intensity was quantified by densitometry using ImageJ software (NIH, Bethesda, MA, United States).

### Immunofluorescence Staining

The cells were cultured on collagen-coated coverslips for immunofluorescence staining. Cells were rinsed three times with cold DPBS with Ca^2+^ and Mg^2+^ and fixed with 2% paraformaldehyde for 10 min. The cells were then incubated with blocking solution (2% donkey serum, 2% fish skin gelatin, 1% BSA, and 0.2% Triton X-100 in PBS) for 1 h, followed by incubation with affinity-purified polyclonal Cx43 antibody (1:100) at 4°C overnight (Cherian et al., 2003). The primary antibody was detected using 1:500 dilution of Alexa Fluor 488- or Alexa Fluor 594–conjugated donkey anti-rabbit antibody (Jackson Immuno Research Labs, 711-545-152 or 711-585-152, West Grove, PA, United States) for 1 h. Cells on coverslips were mounted using the Vectashield mounting medium (Vector Laboratories, H-1000, Burlingame, CA, United States) and sealed. Fluorescence imaging was performed using a confocal laser scanning microscope (Zeiss, LSM780, Jena, Germany) or a fluorescent microscope (Keyence, BZ-X710, Osaka, Japan).

### Scrape Loading/Dye Transfer Assay

Gap junction intercellular coupling was determined at 25°C using scrape loading/dye transfer technique in undifferentiated confluent ROSA26 or Cx43 KD IDG-SW3 cells (Hua et al., 2021). In brief, cells were washed twice with DPBS containing Ca^2+^ and Mg^2+^, and scrape-loading was performed by scraping cells with a sharp razor scraper containing the gap junction–permeable fluorescent dye (1% lucifer yellow, 457 Da) and gap junction–non-permeable fluorescent dye (1% rhodamine–dextran, 10 kDa). After 5 min, cells were washed four times with DPBS and then fixed with 2% paraformaldehyde for 10 min. Fluorescence images were captured using an inverted fluorescent microscope (Olympus IX70, Tokyo, Japan). Experiments were repeated three times, and data were quantified by averaging fluorescence areas of three fields using NIH ImageJ software. Quantification of changes in dye coupled under different groups was performed by measuring the fluorescence area in square millimeters of the lucifer yellow fluorescence minus rhodamine-–dextran fluorescence.

### Fluid Flow Shear Stress (FFSS)

FFSS experiment was conducted to apply mechanical stimulation on undifferentiated ROSA26 or Cx43 KD IDG-SW3 cells. As described previously (Cheng et al., 2001; Riquelme et al., 2015), fluid flow was generated by using a parallel plate flow chamber system (Bioptechs, Butler, PA, United States). The chambers were separated by a gasket of defined thickness with gravity-driven fluid flow using a peristaltic pump (Cole-Parmer Instrument, Chicago, IL, United States). The wall shear stress experienced by cells in these chambers was related directly to the flow rate of the circulating medium through the channel and inversely to the square of the channel height. By adjusting the channel height and flow rate, stress levels of 16 dyn/cm^2^ were established. Cells were cultured on collagen-coated microscope glass slides, which can be mounted on the flow chamber with the surface area of 5 cm^2^ for shear stress exposure. Each test was conducted for 10 min. The circulating medium was recording media (HCO^3−^-free α-MEM medium buffered with 10 mM HEPES, pH 7.4), and controls are consisted of ROSA26 or Cx43 KD IDG-SW3 cells in recording media but not subjected to FFSS.

### Dye Uptake Assay

On completion of the flow regimen, the cell-covered slides were removed for the dye uptake assay. Cells were incubated with a mixture of 0.1 mM ethidium bromide (EtBr, MW 394 Da) and 1 mg/ml FITC-dextran (MW 10 kDa) for 5 min. EtBr was used as a tracer to detect hemichannel activity, and FITC-dextran, which is too large to pass through hemichannels but is taken up by dying cells, was used as a negative control. Cells were then rinsed five times with PBS, followed by fixing with 2% paraformaldehyde for 10 min. At least six microphotographs of fluorescence fields were captured under a 20X fluorescent microscope (Keyence, BZ-X710, Osaka, Japan). For each image, the average intensity of EtBr fluorescence was measured and quantified from at least 30 random cells using ImageJ software (NIH, Bethesda, MD, United States). Experiments were repeated three times, and the collected data were illustrated as pixel mean in arbitrary units.

### RNA Isolation and Real-Time PCR

Total RNA was isolated from differentiated IDG-SW3 cells using the TRIzol reagent (Molecular Research Center, TR118, Cincinnati, OH, United States), according to the manufacturer’s instructions. After RNA quantification by using Nanodrop 2000, cDNA was synthesized from 1 μg of total RNA using the high-capacity RNA-to-cDNA kit (Applied Biosystems, 43-889-50, Bedford, MA, United States). Real-time PCR was performed using an ABI 7900 PCR device (Thermo Fisher Scientific) and SYBR Green (Bio-Rad Laboratories, 1725124, Hercules, CA, United States) with a two-step protocol (94°C for 15 s and 60°C for 60 s). The 2^−ΔΔCT^ method was used for qPCR data analysis. GAPDH was used as a housekeeping gene control. The primers sequences used in this study are listed in Table 1. Experiments were run in triplicates.

**TABLE 1:** Primers sequences for RT-PCR.

Gene	Forward primer (5′–3′)	Reverse primer (5′–3′)
ALP	GGA​GAT​GGA​CCA​GGC​CAT​TG	CCG​TCC​ACC​ACC​TTG​TAG​CC
COL1A1	GCC​AAT​GGT​GCT​CCT​GGT​ATT​G	TTT​GGC​ACC​AGT​GTC​TCC​TTT​G
DMP1	CCC​AGT​TGC​CAG​ATA​CCA​CAA​TAC	GCT​GTC​CGT​GTG​GTC​ACT​ATT​T
SOST	CAT​CCC​AGG​GCT​TGG​AGA​GTA	TGT​CAG​GAA​GCG​GGT​GTA​GT
FGF23	CTA​CAG​CCA​GGA​CCA​GCT​ATC​A	GTT​GCC​GTG​GAG​ATC​CAT​ACA​AAG
OPG	GAA​TGC​CGA​GAG​TGT​AGA​GAG​GAT​AA	CGC​TGC​TTT​CAC​AGA​GGT​CAA​T
RANKL	CCG​TGC​AGA​AGG​AAC​TGC​AA	TAT​GGG​AAC​CCG​ATG​GGA​TG

### Alkaline Phosphatase, Alizarin Red, and *von Kossa* Staining

For alkaline phosphatase staining, after osteogenic induction for 9 days, cells were fixed with 4% paraformaldehyde for 10 min and incubated in freshly prepared naphthol AS-MX phosphate (Sigma-Aldrich, 855) and Fast Blue BB (Sigma-Aldrich, F0500) solution. For Alizarin red staining of calcium, after osteogenic induction for 18 or 28 days, cells were fixed with 10% buffered formalin for 15 min and then stained with 2% Alizarin red solution, pH 4.2 (Sigma-Aldrich, A5533). For *von Kossa* staining of phosphate, after osteogenic induction for 28 days, cells were fixed with 10% buffered formalin for 15 min. Cells were then incubated with 5% silver nitrate solution under ultraviolet (UV) light for 20 min. Images were captured using a Keyence microscope (BZ-X710, Osaka, Japan). The stained area was calculated as a percent of total well area over a representative threshold level.

### Conditioned Medium Treatment and Tartrate-Resistant Acid Phosphatase (TRAP) Staining

RAW264.7 osteoclast precursors were grown in the RPMI-1640 medium (Thermo Fisher Scientific) supplemented with 10% FBS and 1% penicillin/streptomycin. The conditioned medium collected from differentiated IDG-SW3 cells was aliquoted and stored at −80°C before use. RAW264.7 cells were seeded in 48-well plates for osteoclastogenesis induction. RAW264.7 cells were supplied with the conditioned medium and RAW264.7 growth media mixed in the ratio of 1:1, with 10 ng/ml or 50 ng/ml recombinant RANKL (R&D Systems, 462-TEC-010, Minneapolis, MN, United States). After 7 days of differentiation, osteoclasts were visualized using a leukocyte acid phosphatase staining kit (Sigma, 387A-1 KT). Images were captured using a Keyence microscope (BZ-X710, Osaka, Japan).

### Statistical Analysis

Statistical analysis was performed using GraphPad Prism8 statistics software (GraphPad, San Diego, United States). All data are presented as mean ± SEM. *t*-test, one-way ANOVA, and two-way ANOVA with Tukey’s test was used for statistical analysis. Asterisks indicate the degree of significant differences compared with the controls (*, *p* < 0.05; **, *p* < 0.01; ***, *p* < 0.001; ****, *p* < 0.0001).

## Results

### Cx43 Expression is Increased During Osteogenic Differentiation

**FIGURE 1:**
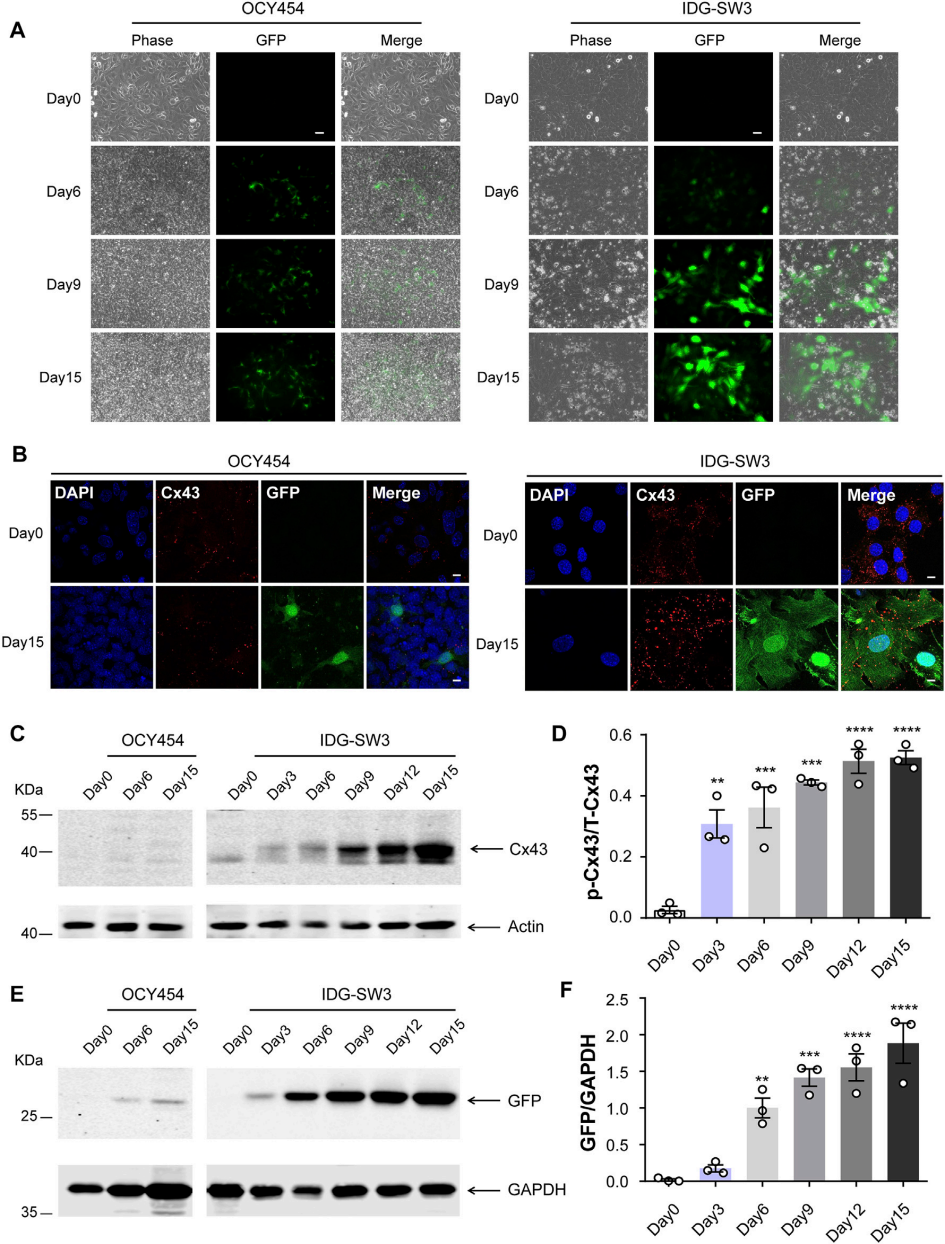
Cx43 expression is increased during osteogenic differentiation in DMP1-GFP–expressing IDG-SW3 and OCY454 cells **(A)** Representative images of DMP1-GFP expression in OCY454 (left panels) and IDG-SW3 cells (right panels) under fluorescent and phase-contrast microscopy during the time course from immortalizing (Day 0) to osteogenic differentiation (Day 15). Scale bar, 50 μm. **(B)** Immunofluorescence staining of Cx43 in OCY454 (left panels) and IDG-SW3 cells (right panels) under immortalizing (Day 0) and osteogenic differentiation (Day 15) conditions. Nuclei were stained with DAPI (blue). Scale bar, 10 μm. **(C)** Membrane extracts were prepared from OCY454 and IDG-SW3 cells and subjected to immunoblotting using Cx43 (CT) or β-actin antibodies. **(D)** Densitometry measurement ratio of phosphorylated Cx43 (p-Cx43) to total Cx43 (T-Cx43). **(E)** Soluble protein extract was isolated from OCY454 and IDG-SW3 cells and subjected to immunoblotting using GFP or GAPDH antibodies. **(F)** Densitometry measurement ratios of GFP to GAPDH. Data shown are mean ± SEM. **, p < 0.01, ***, p < 0.001, ****, p < 0.0001.

OCY454 and IDG-SW3 cell lines are two cell models of differentiated osteocytes, which are derived from long bones of double transgenic mice expressing DMP1-GFP and thermolabile large T antigen that enables conditional immortalization of cells (Woo et al., 2011; Spatz et al., 2015). At 33°C, OCY454 and IDG-SW3 cells showed continuous proliferation and were GFP-negative (Figure 1A, Day 0). After osteogenic induction, they exhibited late osteoblast to osteocyte phenotype, with expression of a DMP1-GFP reporter as a marker for osteocytic differentiation (Figure 1A, Day 6–Day 15). The percentage of GFP-positive cells increased along with the differentiation process in both cell lines. Cx43 expression in OCY454 and IDG-SW3 cells were examined by immunofluorescence staining (Figure 1B). Under proliferating conditions, the subcellular localization of Cx43 was mainly in the cytoplasmic region, which is consistent with a previous report in osteoblasts (Yang et al., 2021). The differentiated IDG-SW3 cells displayed a clustered punctate spot-like distribution of Cx43 toward the cell surface (Figure 1B, right panels). However, the difference of Cx43 distribution patterns between proliferating and differentiated OCY454 cells was not evident (Figure 1B, left panels). To further quantitatively evaluate the Cx43 expression level, Western blot was performed using crude membrane extracts isolated from both cell lines at different time point of differentiation. As shown in Figure 1C, there was a minimal level of Cx43 in proliferating OCY454 cells revealed by the affinity-purified Cx43 antibody. In addition, Cx43 expression in differentiated OCY454 cells is very low. In contrast, the amount of Cx43 protein increased dramatically in IDG-SW3 cells along with the differentiation process. The posttranslational phosphorylation of Cx43 was increased associated with differentiation of IDG-SW3 cells (Figure 1C), showing the band with decreased electrophoretic mobility due to the addition of phosphate (Musil and Goodenough, 1991; Cheng et al., 2001). The quantification of the phosphorylated Cx43 (p-Cx43)/total Cx43 (T-Cx43) ratio showed a marked elevation during differentiation (Figure 1D). We next probed GFP expression in soluble protein prepared at different time point of differentiation. In Figure 1E, GFP was increased in differentiated OCY454 cells compared to proliferation condition. However, GFP increase was more profound in IDG-SW3 cells during differentiation. The changes can also be reflected in the densitometry analysis for the GFP level (Figure 1F). Taken together, during the osteogenic differentiation process, Cx43 protein was significantly increased and differentiated IDG-SW3 cells expressed more Cx43 than OCY454 cells.

### Generation of Cx43 Knockdown IDG-SW3 Cells Using CRISPR/Cas9 Genome Editing Technology

**FIGURE 2:**
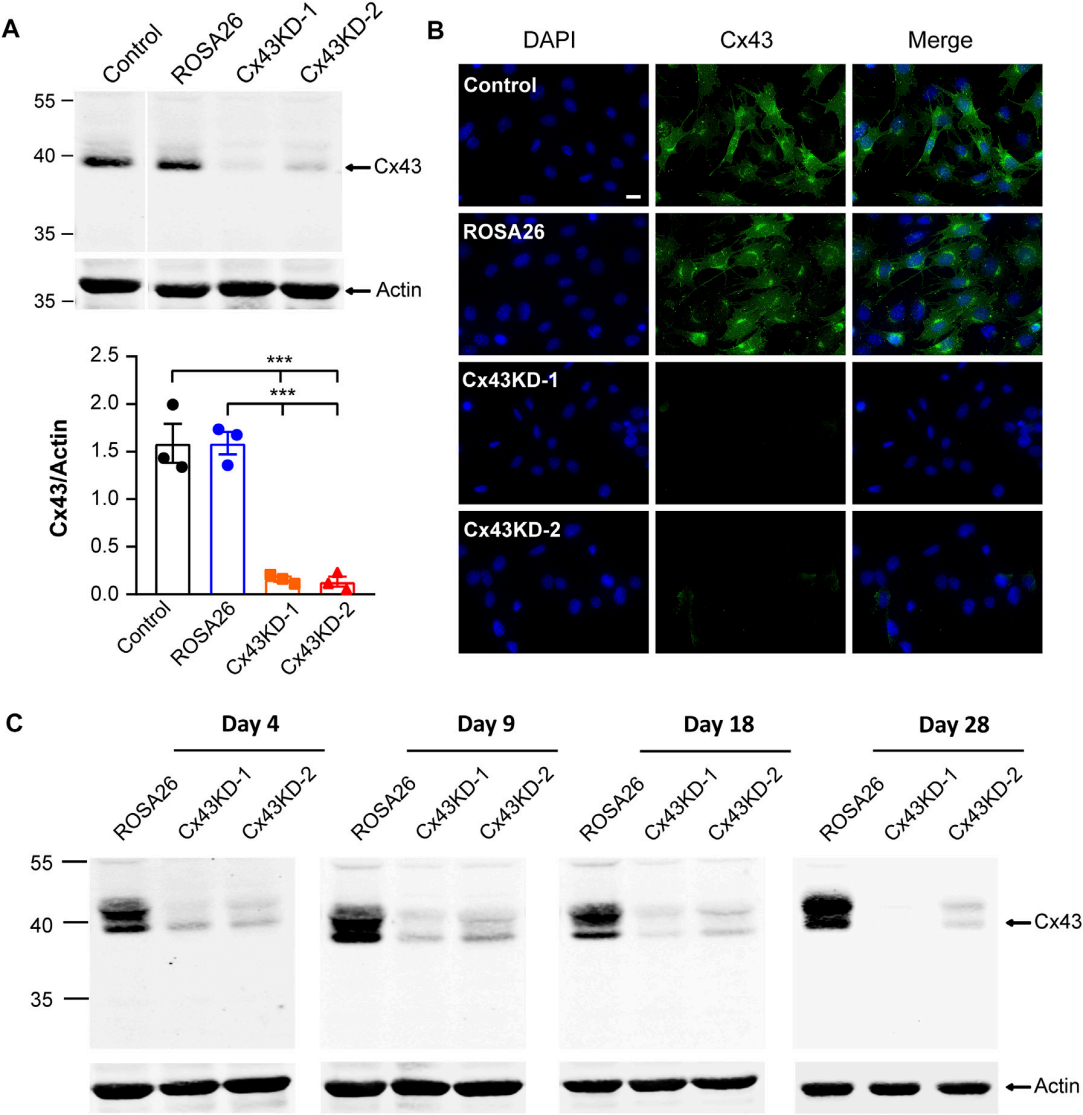
Ablation of Cx43 expression using CRISPR/Cas9 gene editing technology in IDG-SW3 cell line. **(A)** Immortalizing (Day 0) IDG-SW3 cells were subjected to specified ROSA26 or Cx43-sgRNA lentivirus transduction and puromycin selection. Membrane extracts were immunoblotted by Cx43 or β-actin antibodies (upper panel). The lower panel shows the densitometric measurement ratios of Cx43 to β-actin. **(B)** Representative images of Cx43 immunofluorescence staining in control, ROSA26, or Cx43 KD IDG-SW3 cells. Scale bar, 20 μm. **(C)** Membrane extracts were isolated from ROSA26 or Cx43 KD groups during the time course from Day 4 to Day 28 after differentiation. Cx43 expression was analyzed by using Western blot probed with Cx43 and β-actin antibodies. Data shown are mean ± SEM. ***, p < 0.001.

We next focused on the IDG-SW3 cells to study the role of Cx43 in regulating osteoblast to osteocyte differentiation process. Using lentiviral-mediated CRISPR/Cas9 genome editing technology, we generated Cx43 KD cells after lentivirus infection of two pairs of sgRNA followed by puromycin selection. ROSA26, as a preferred site for the integration of transgenes and reporter constructs, is ubiquitously expressed in all cell types and developmental stages (Irion et al., 2007; Chu et al., 2016). In our study, sgRNA targeting ROSA26 was designed for lentivirus infection and puromycin selection in IDG-SW3 cells, which could serve as a reference control (Bäck et al., 2019; Riggan et al., 2020). The expression of Cx43 was determined by Western blot. There was a remarkably band intensity reduction after CRISPR/Cas9-mediated ablation of Cx43, and the Cx43 level is comparable between ROSA26 and non-targeting control groups (Figure 2A, upper panel). Quantification of the Cx43/actin ratio showed a 90% decrease in Cx43 KD groups compared to ROSA26 or control (Figure 2A, lower panel). The immunofluorescence staining further validated the successful generation of Cx43 KD strains in proliferating IDG-SW3 cells (Figure 2B). During the osteoblast to osteocyte transition, IDG-SW3 cells undergo several differentiation stages: early osteoblast (day 4), osteoid osteocyte (day 9), mineralizing osteocyte (day 18), and mature osteocyte (day 28) (Woo et al., 2011). We cultured IDG-SW3 cells from ROSA26 and Cx43 KD groups for differentiation to examine whether the CRISPR/Cas9-mediated Cx43 deficiency could be maintained throughout the differentiation process. Membrane extracts collected at different differentiation stages were subjected to Western blot analysis. As shown in Figure 2C, compared to the ROSA26 group, Cx43 KD groups had minimal Cx43 expression at each time point. These results demonstrated the generation of stable Cx43 KD IDG-SW3 cell lines by using lentiviral-mediated CRISPR/Cas9 genome editing technology.

### Cx43 Knockdown in IDG-SW3 Cells Inhibits Gap Junction Intercellular Communication and the Opening of Cx43 Hemichannels

**FIGURE 3:**
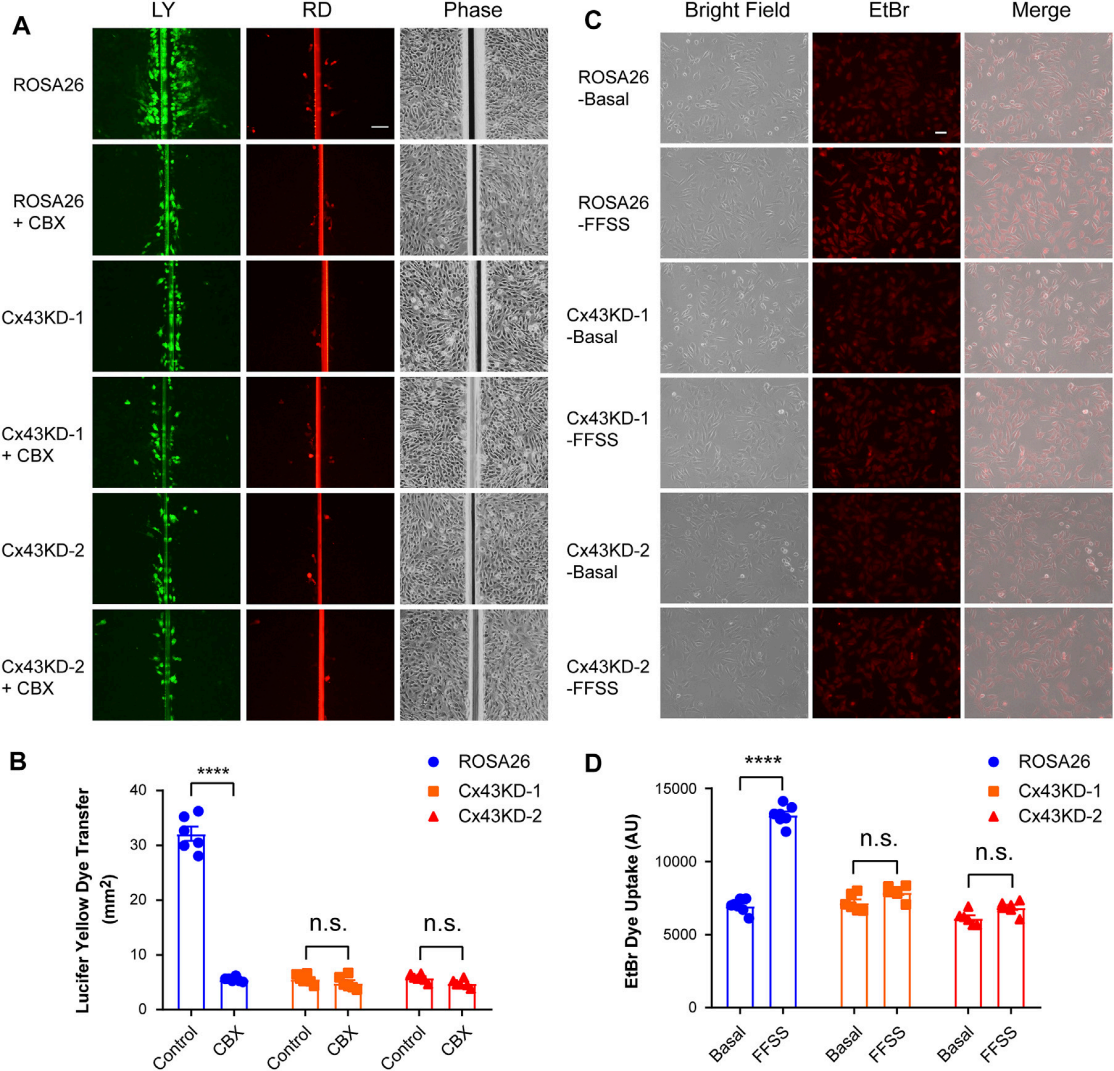
Cx43 deficiency in IDG-SW3 cell line inhibits gap junction intercellular communication and the opening of Cx43 hemichannels. **(A)** Dye transfer was determined in ROSA26 or Cx43 KD IDG-SW3 cells in absence or presence of 100 μM CBX. The scrape loading assay was performed with LY and RD for 5 min. Scale bar, 200 μm. **(B)** Area of dye transfer was determined and quantified by fluorescence microcopy and NIH ImageJ software. **(C)** ROSA26 or Cx43 KD IDG-SW3 cells were subjected to FFSS for 10 min or non-FFSS basal conditions, followed by incubation with 100 μM EtBr for 5 min. Scale bar, 50 μm. **(D)** Level of EtBr dye uptake was determined and quantified by fluorescence microcopy and NIH ImageJ software. Data shown are mean ± SEM. ****, p < 0.0001.

The scrape loading/dye transfer assay is a commonly used approach to study intercellular coupling *via* functional gap junction channels (el-Fouly et al., 1987). Gap junction intercellular communication (GJIC) was evaluated in undifferentiated ROSA26 and Cx43 KD IDG-SW3 cells. Cells were grown to reach confluence before performing the scrape loading/dye transfer assay. As illustrated in Figure 3A and quantified in Figure 3B, the ROSA26 group was much more efficient in transferring LY in comparison with Cx43 KD groups. Carbenoxolone (CBX) acts as a potent and effective blocker of GJIC (Connors, 2012). After CBX inhibition, the ROSA26 group showed decreased intercellular coupling compared to that of the Cx43 KD level (Figure 3A,B). There was no difference between Cx43 KD IDG-SW3 cells with or without CBX blocking, indicating the CRISPR/Cas9-mediated Cx43 deficiency inhibited GJIC. The activity of Cx43 HCs was further evaluated using the EtBr dye uptake assay. Cells were grown at a low-cell density to ensure that the majority of the cells were not physically in contact. Our previous study has demonstrated that Cx43 HCs open when subjected to mechanical stimulation in the form of FFSS (Cherian et al., 2005). Under FFSS, the ROSA26 group showed a 2-fold increase in arbitrary units (AU) of fluorescence intensity, reflective of the hemichannel dye uptake level, compared to that of the basal level (Figure 3C,D). In contrast, Cx43 KD groups were not responding to FFSS-induced HCs opening, indicating an impaired Cx43 HCs activity in undifferentiated IDG-SW3 cells with Cx43 KD.

### Cx43 Knockdown in IDG-SW3 Cells Decreases Alkaline Phosphatase and Type I Collagen Expression at Early Differentiation Stage

**FIGURE 4:**
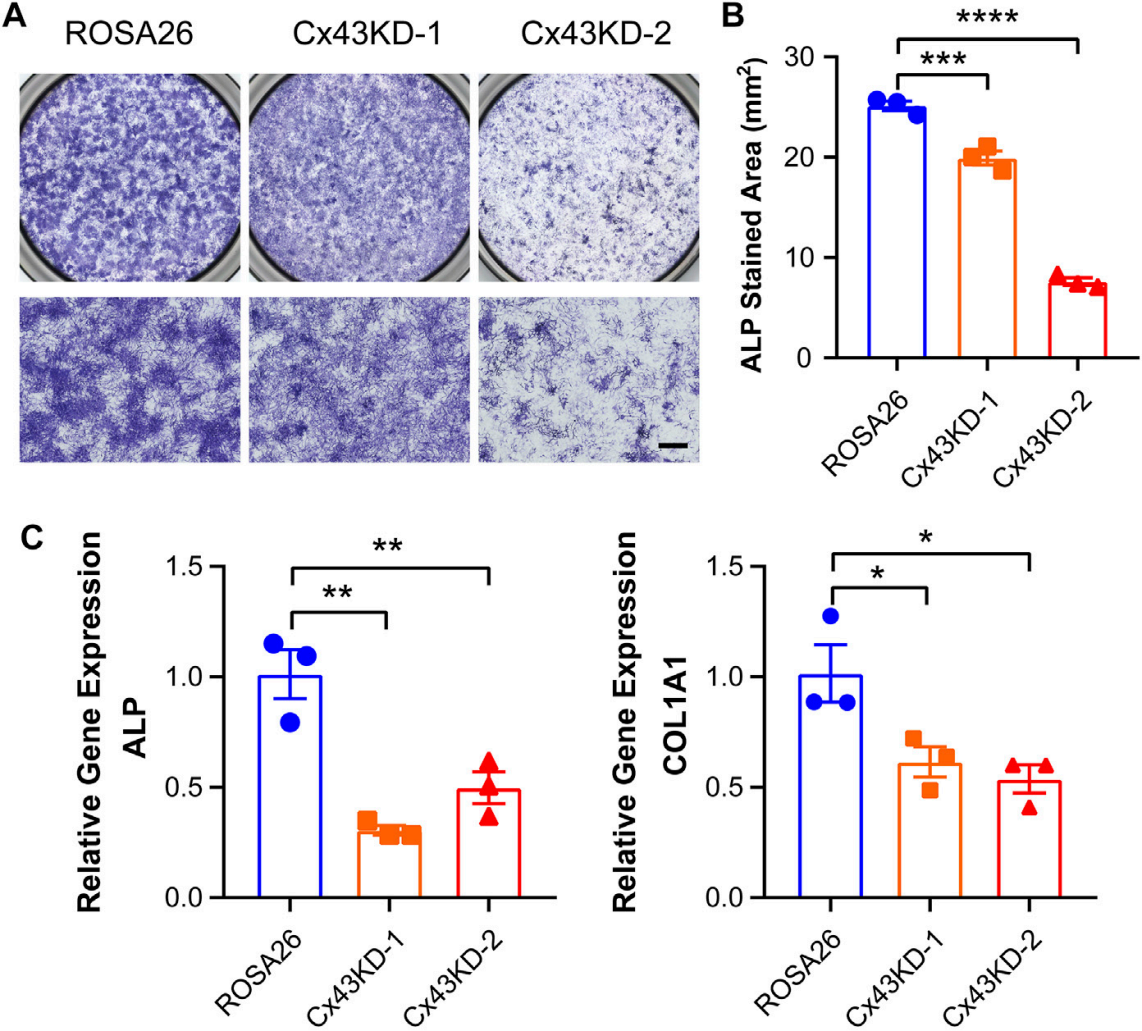
Cx43 knockdown decreased alkaline phosphatase and type I collagen expression in IDG-SW3 cells. **(A)** Representative images of ALP staining in ROSA26 or Cx43 KD IDG-SW3 cells at day 9 of the induction of differentiation. Lower panels show an enlarged view of the center regions of upper panels. Scale bar, 500 μm. **(B)** Quantification of ALP-stained area by NIH ImageJ software. **(C)** Total RNA was prepared from ROSA26 or Cx43 KD IDG-SW3 cells. The mRNA levels of ALP and COL1A1 were determined by real-time PCR normalized to GAPDH. Data shown are mean ± SEM. *, p < 0.05, **, p < 0.01, ***, p < 0.001, ****, p < 0.0001.

IDG-SW3 cells express osteoblastic markers including alkaline phosphatase (ALP) and type I collagen (COL1A1) at the early stage of differentiation. To evaluate the osteoblastic activity of ROSA26 and Cx43 KD IDG-SW3 cells, ALP staining was performed after 9 days of differentiation. Cx43 KD groups had significantly reduced the level of positive ALP staining (Figure 4A,B). Using real-time PCR, we also observed lower abundance of ALP and COL1A1 mRNA with Cx43 deficiency (Figure 4C), further suggesting that Cx43 KD in IDG-SW3 cells decreases osteoblastic marker expression.

### Cx43 Knockdown in IDG-SW3 Cells Decreases Mineralization and Osteocytic Genes Expression at Late Differentiation Stage

**FIGURE 5:**
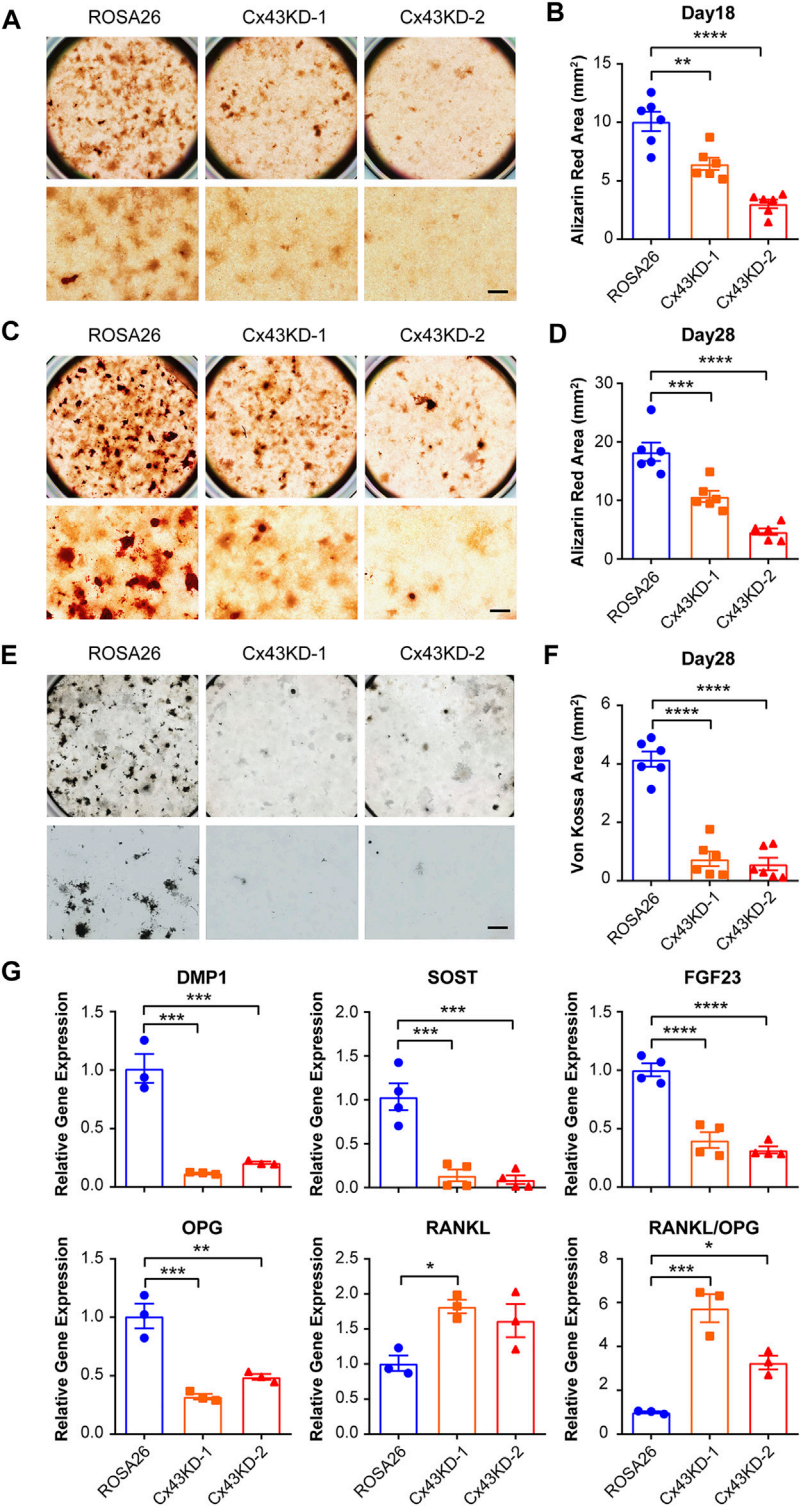
Cx43 knockdown decreased matrix mineralization and osteocytic markers expression in IDG-SW3 cells. Representative images of Alizarin red staining in ROSA26 or Cx43 KD IDG-SW3 cells at day 18 **(A)** or 28 **(C)** of the induction of differentiation. Lower panels show an enlarged view of the center regions of upper panels. Scale bar, 500 μm. **(B,D)** Quantification of Alizarin red–stained area by NIH ImageJ software. **(E)** Representative images of *von Kossa* staining in ROSA26 or Cx43 KD IDG-SW3 cells after 28 days of differentiation. Lower panels show an enlarged view of the center regions of upper panels. Scale bar, 500 μm. **(F)** Quantification of *von Kossa*-stained area by NIH ImageJ software. **(G)** Total RNA was prepared from ROSA26 or Cx43 KD IDG-SW3 cells after 28 days of differentiation. The mRNA levels of DMP1, SOST, FGF23, OPG, and RANKL were determined by real-time PCR normalized to GAPDH. Data shown are mean ± SEM. *, p < 0.05, **, p < 0.01, ***, p < 0.001, ****, p < 0.0001.

We then tested whether Cx43 plays a major role in mineralization and calcium deposition during osteogenic differentiation. ROSA26 and Cx43 KD IDG-SW3 cells were cultured in a mineralizing medium containing ascorbic acid and β-glycerophosphate for 18 and 28 days. Alizarin red staining for calcium deposition showed that the staining area was substantially lower in Cx43 KD groups than that in ROSA26 cells at both 18 days (Figure 5A,B) and 28 days (Figures 5C,D) after differentiation. In addition, *von Kossa* staining for focal nodular mineralization was performed at 28 days. The number of mineralized nodules was reduced by 80% in IDG-SW3 cells with Cx43 KD (Figure 5E,F). The mRNA expression of DMP1, SOST, FGF23, osteoprotegerin (OPG), and receptor activator of nuclear factor kappa-B ligand (RANKL) was determined. After 28 days of osteogenic differentiation, DMP1, SOST, FGF23, and OPG showed significant decrease in both Cx43 KD groups compared to that in the ROSA26 group (Figure 5G). In contrast, the level of RANKL mRNA was elevated up to almost 2-fold in Cx43 KD IDG-SW3 cells. Correspondingly, the ratio of RANKL/OPG, which is indicative of the propensity to generate osteoclasts during bone remodeling (Boyce and Xing, 2008), was significantly elevated in IDG-SW3 cells with Cx43 KD (Figure 5G). The aforementioned results indicated delayed osteogenic differentiation caused by Cx43 deficiency, along with a decrease in DMP1, SOST, and FGF23, and an increase in the RANKL/OPG ratio.

### Blocking Cx43 Hemichannels in IDG-SW3 Cells Reduces Osteoblastic and Osteocytic Genes Expression During Differentiation

**FIGURE 6:**
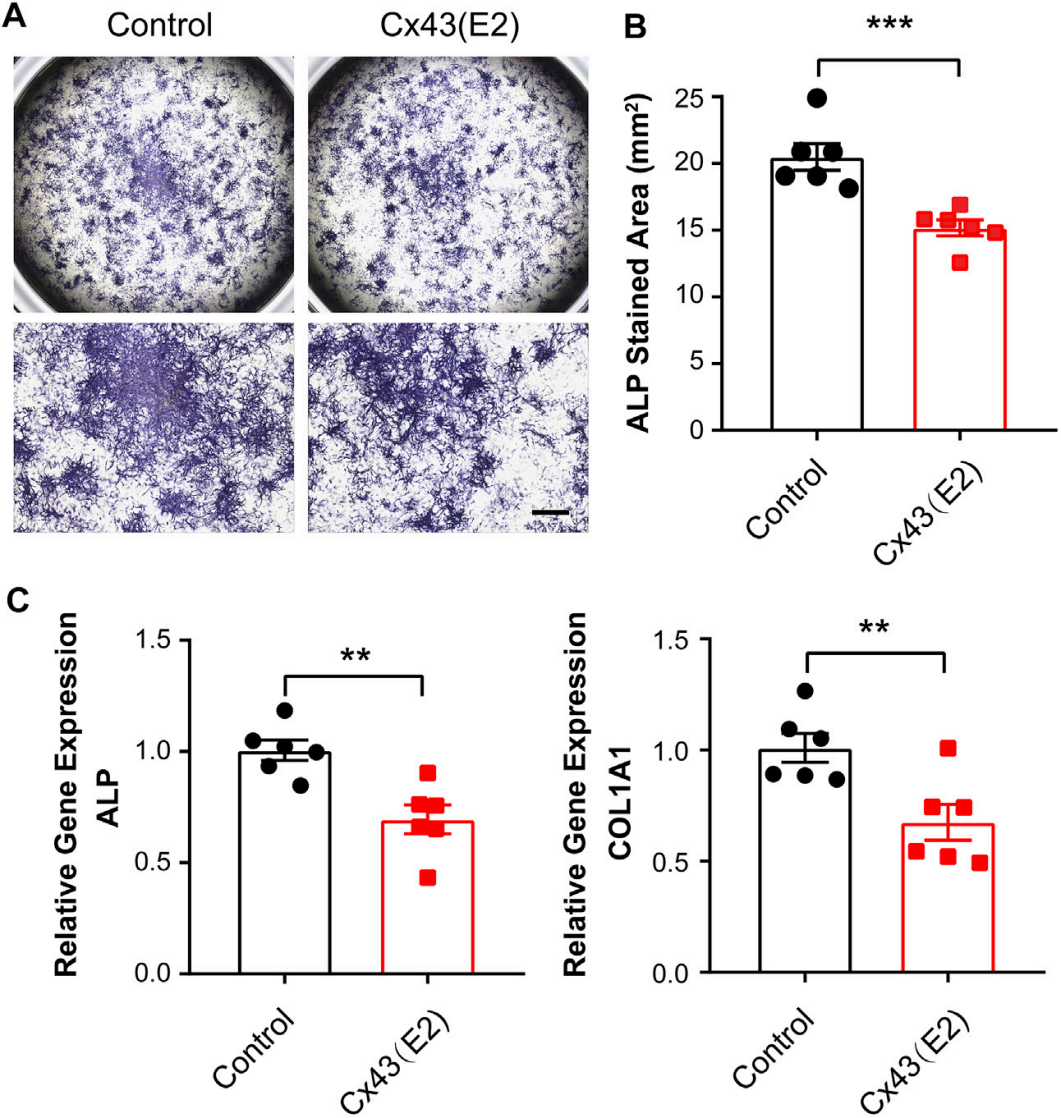
Inhibition of Cx43 hemichannels decreased alkaline phosphatase and type I collagen expression in IDG-SW3 cells. **(A)** Representative images of ALP staining in control or Cx43 (E2) antibody–treated IDG-SW3 cells at day 9 of the induction of differentiation. Lower panels show enlarged view of the center regions of upper panels. **(B)** Quantification of ALP-stained area by NIH ImageJ software. **(C)** Total RNA was prepared from control or Cx43 (E2) antibody–treated IDG-SW3 cells. The mRNA levels of ALP and COL1A1 were determined by real-time PCR normalized to GAPDH. Data shown are mean ± SEM. **, p < 0.01, ***, p < 0.001.

We then assessed whether Cx43 HCs could play a role in regulating the osteoblastic genes expression. To specifically target the Cx43 HCs, we utilized the Cx43 (E2) antibody, which was previously generated by our lab (Siller-Jackson et al., 2008). This blocking antibody targets the second extracellular loop (E2) of Cx43. IDG-SW3 cells were treated with or without Cx43 (E2) antibody for 9 days during differentiation. The ALP-stained area was significantly decreased in Cx43 (E2)-treated cells (Figure 6A,B). Consistently, ALP and COL1A1 mRNA levels were much lower in the Cx43 (E2) group than those in the control group (Figure 6C).

**FIGURE 7:**
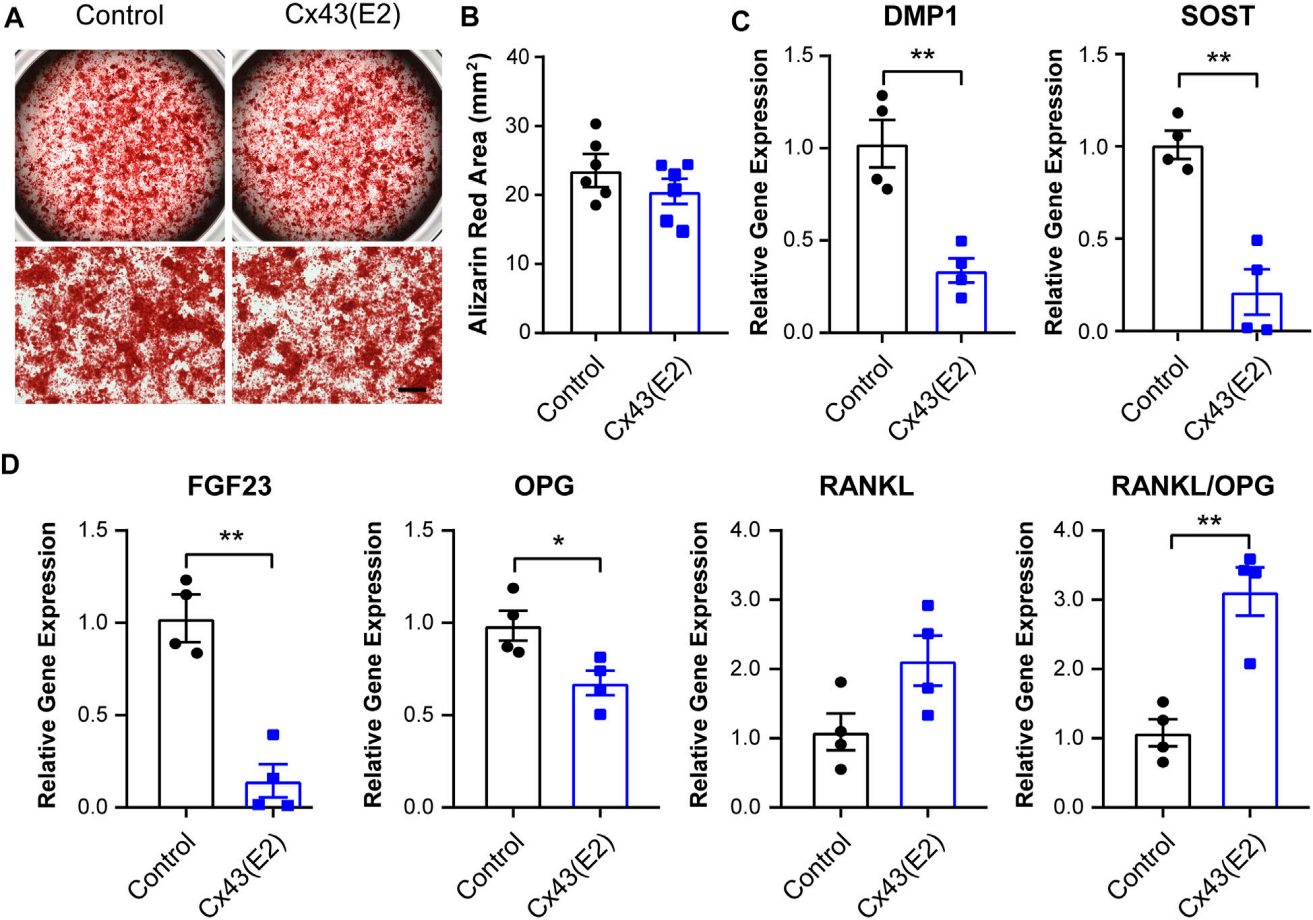
Inhibition of Cx43 hemichannels decreased osteocytic markers expression in IDG-SW3 cells. **(A)** Representative images of Alizarin red staining in control or Cx43 (E2) antibody–treated IDG-SW3 cells at day 28 of the induction of differentiation. Lower panels show enlarged view of the center regions of upper panels. Scale bar, 500 μm. **(B)** Quantification of Alizarin red–stained area by NIH ImageJ software. **(C–D)** Total RNA was prepared from control or Cx43 (E2) antibody–treated IDG-SW3 cells after 28 days of differentiation. The mRNA levels of DMP1, SOST, FGF23, OPG, and RANKL were determined by real-time PCR normalized to GAPDH. Data shown are mean ± SEM. *, p < 0.05, **, p < 0.01.

To study the role of Cx43 HCs in the regulation of mineralization and osteocytic marker genes expression, we incubated IDG-SW3 cells with Cx43 (E2) antibody for 28 days during differentiation. There was no significant difference of calcium deposition evaluated by Alizarin red–stained area after Cx43 (E2) treatment (Figure 7A,B). The mRNA expression of DMP1, SOST, FGF23, OPG, and RANKL was also determined. The Cx43 (E2) antibody–treated IDG-SW3 cells showed significant downregulation of DMP1, SOST, FGF23, and OPG. With the Cx43 (E2) inhibition of Cx43 HC activity, the RANKL mRNA exhibited a trend of elevation, resulting in a significantly increased RANKL/OPG ratio compared to the control group.

### Conditioned Medium From Cx43 Knockdown IDG-SW3 Cells Promotes Osteoclastogenesis of RAW264.7 Cells

**FIGURE 8:**
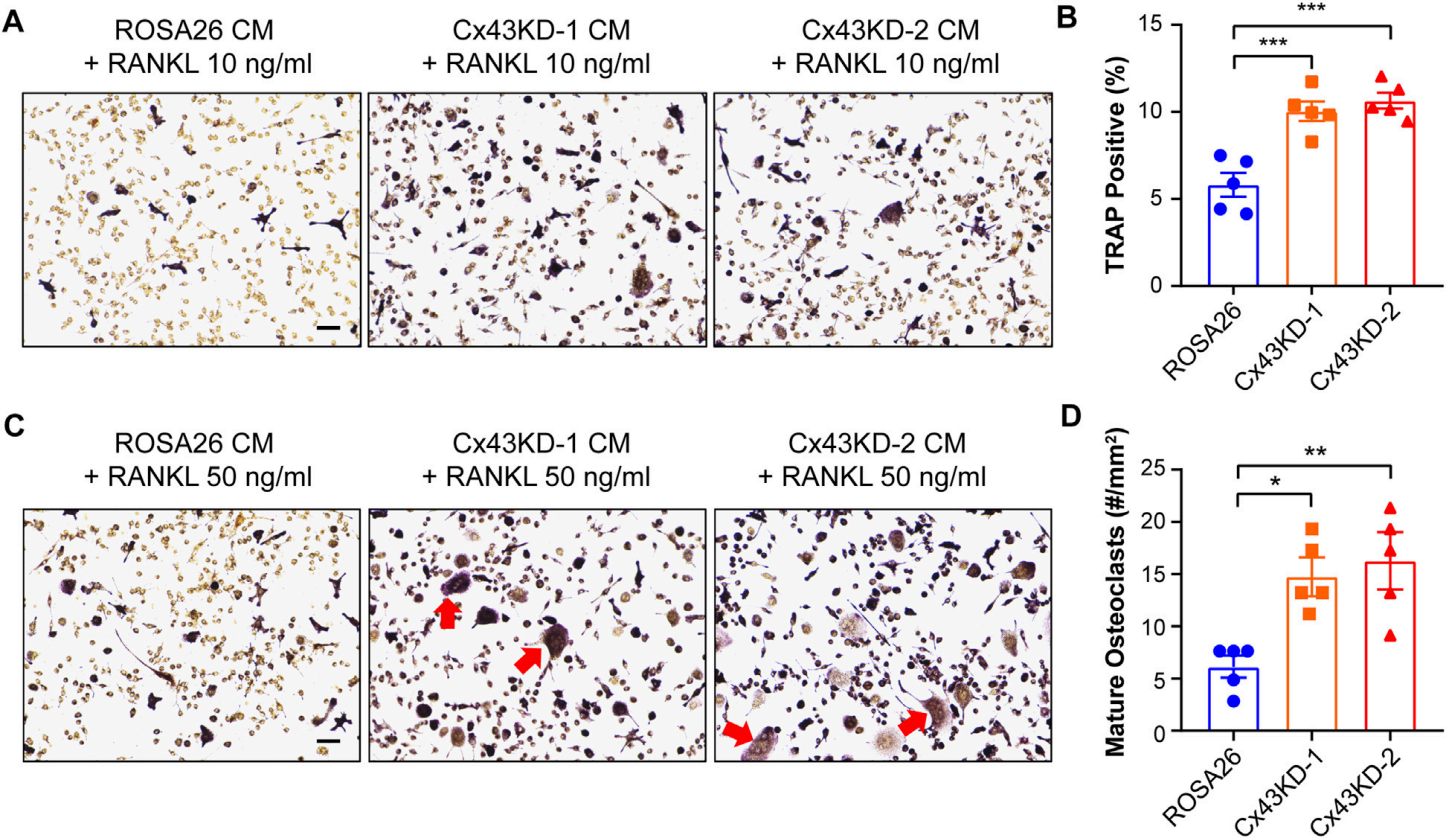
Conditioned medium collected from differentiated IDG-SW3 cells with Cx43 knockdown promoted osteoclastogenesis of RAW264.7 cells. Representative images **(A,C)** of TRAP staining in differentiated RAW264.7 cells cultured in CM from ROSA26 or Cx43 KD IDG-SW3 cells with 10 ng/ml **(A,B)** or 50 ng/ml RANKL **(C,D)** for 7 days. Scale bar, 50 μm. **T**he percentage of TRAP-stained cells **(B)** and numbers of mature osteoclasts **(D)** were quantified by NIH ImageJ software. Data shown are mean ± SEM. *, p < 0.05, **, p < 0.01, ***, p < 0.001.

Osteocytes express essential bone modulating factors which could act directly to regulate osteoclast formation and bone resorption (Kitaura et al., 2020). Given the impaired osteogenesis function under Cx43 deficiency condition, we next investigated whether the secreted factors from ROSA26 and Cx43 KD osteocytes could influence osteoclastogenesis. The conditioned medium (CM) was collected from IDG-SW3 cells with or without Cx43 deficiency after 28 days of differentiation. RAW264.7 osteoclast precursors were cultured using CM supplemented with 10 ng/ml or 50 ng/ml RANKL for 1 week. As shown in Figure 8A,B, there was a 1.7-fold elevation in TRAP-positive cell percentage in RAW264.7 cells treated with Cx43 KD CM and 10 ng/ml RANKL. The treatment with Cx43 KD CM and 50 ng/ml RANKL showed more large osteoclasts with multi-nuclei (Figure 8C, red arrows), and quantification further demonstrated a significant increase of mature osteoclasts numbers compared to the ROSA26 control (Figure 8D). These results indicate that Cx43 channels in IDG-SW3 cells may secrete factors that inhibit osteoclast differentiation.

## Discussion

In this study, we found that during IDG-SW3 cell differentiation process, Cx43 expression increased dramatically, and their Cx43 expression level is much higher than OCY454 cells. We effectively deleted Cx43 in IDG-SW3 cell model using lentiviral-based CRISPR/Cas9 genome editing technique. IDG-SW3 cells with Cx43 deficiency exhibited decreased osteoblast markers, ALP and COL1A1, expression at the osteoid–osteocyte stage compared to control. With the progression from mineralization to maturation of osteocytes, Cx43 deficiency resulted in a reduction in matrix-forming protein—DMP1, mature osteocyte markers—SOST and FGF23, and a significant increase in the RANKL/OPG level. Moreover, blocking Cx43 HCs using a specific Cx43 antibody resulted in downregulation of ALP, COL1A1, DMP1, SOST, and FGF23, with an increase of RANKL/OPG. Interestingly, RAW264.7 osteoclast precursors were more prone to osteoclastogenesis after treatment with CM from Cx43 KD IDG-SW3 cells. Our findings suggest that Cx43 HCs plays an essential role in osteoblast to osteocyte differentiation and mineralization process as well as in regulating osteoclast differentiation *via* secreted factors.

The CRISPR/Cas9 system is a fast and efficient tool to conduct genomic modification (Wang et al., 2018). In combination with the lentivirus infection, the sgRNA and Cas9 sequences can be integrated into the genome of target cells. Our results showed that the lentiviral-mediated CRISPR/Cas9 expression was steadily maintained, which can continuously exert excision functions throughout the IDG-SW3 cell differentiation process. Cx43 consists of two exons: exon1 encodes most of the 5'-untranslated region (5'-UTR) and exon2 contains complete coding sequence and 3′-UTR (Pfeifer et al., 2004). To silence the Cx43 gene, two sgRNA oligonucleotides targeting the exon2 were designed and validated to avoid the effects of potential off-target activities. Both pairs of sgRNAs led to remarkable reduction of GJIC and HC opening induced by FFSS in IDG-SW3 cells. The ROSA26 locus is often referred to as a “safe harbor” locus and has been extensively used as a transgene insertion site (Irion et al., 2007; Chu et al., 2016). The sgRNA-targeted ROSA26 gene was constructed and served as a good experimental control. In addition, the validated lentiviral CRISPR/Cas9 vectors could be potentially expanded to application in other cell types.

Cx43 forms gap junctions and HCs, which play essential roles in bone development *in vivo*. The conventional Cx43 knockout mouse model is embryonically lethal, with delayed ossification and craniofacial abnormalities (Lecanda et al., 2000; Chaible et al., 2011). Cx43 conditional deletion models in osteoblasts and/or osteocytes have been developed. The COL1A1 promoter–driven Cx43 deletion results in low bone mineral density (BMD) and compromised bone strength (Chung et al., 2006; Grimston et al., 2008). The human osteocalcin promoter- or DMP1 promoter–driven Cx43 deletion leads to increased osteocytic apoptosis, endocortical bone resorption, and periosteal bone formation (Zhang et al., 2011; Bivi et al., 2012). Studies with osteoblastic cell lines have demonstrated the importance of Cx43 channels in osteoblast proliferation (Gramsch et al., 2001) and in mediating the antiapoptotic effects of bisphosphonates and parathyroid hormone (Plotkin et al., 2002; Bivi et al., 2011). Moreover, Cx43 potentiates osteoblast responsiveness to FGF2 (Lima et al., 2009). Previous reports also reveal the roles of gap junctions in osteoblast differentiation (Jiang et al., 2007). Cx43 expression and gap junction function are shown to increase along with osteoblast differentiation process (Donahue et al., 2000). Overexpression of Cx43 promotes osteoblasts proliferation and differentiation (Lecanda et al., 1998; Gramsch et al., 2001). In contrast, using inhibitors of gap junctions or transfection with antisense Cx43 cDNA/dominant negative Cx43 construct attenuates osteoblast differentiation, which is associated with a reduction in ALP activity, COL1A1 expression, and decreased bone nodule formation (Li et al., 1999; Schiller et al., 2001; Upham et al., 2003). Our study generated CRISPR/Cas9-mediated Cx43 deficiency in a newly developed osteocytic cell model and specifically targeted Cx43 HCs to investigate the regulation from pre-osteocyte to mature osteocyte differentiation. Our results highlighted the unrecognized role of Cx43 HCs in this differentiation process.

To dissect the differential functions of gap junctions vs. HCs formed by Cx43 *in vivo*, we have generated two transgenic mouse models driven by DMP1 promoter with Cx43-dominant negative mutants (Xu et al., 2015). R76W mutant has an impaired gap junction, while both the gap junction and HCs were impaired in Δ130-136 mice. Cx43 HCs play a dominant role in regulating osteocyte survival, endocortical bone resorption, and periosteal apposition. We also showed that functional HCs protect osteocytes against catabolic effects during estrogen deficiency (Ma et al., 2019). In this study, to delineate the role of Cx43 HCs in the regulation of osteoblast to osteocyte differentiation, we adopted the Cx43 (E2) antibody developed in our lab (Siller-Jackson et al., 2008; Riquelme et al., 2013). This polyclonal antibody specifically binds to the E2 domain of Cx43, detects cell surface–expressed Cx43, and impedes the opening of Cx43 HCs, without affecting the docking and function of the gap junction channels. The Cx43 (E2) antibody has been widely used by various groups to determine the function of Cx43 HCs (Bao et al., 2011; Orellana et al., 2012; Tarzemany et al., 2017). Our results demonstrated that blocking Cx43 HCs decreased osteoblastic markers at an early differentiation stage; downregulated DMP1, SOST, and FGF23; and increased RANKL/OPG at the late differentiation stage, which were also observed under Cx43 deficiency condition. These findings indicate that Cx43 HCs play important roles in regulating the differentiation markers expression, possibly through mediating the release of anabolic factors that are elicited during differentiation process. After Cx43 (E2) antibody treatment, the calcium deposition only showed a trend of reduction. One of the possible reasons could be that the matrix accumulated around differentiated cells may impede the accessibility of the antibody to the cell.

RANKL secreted by osteocytes is the key factor for osteoclast formation and bone resorption (Kitaura et al., 2020). OPG acts as a decoy receptor, which protects bone from excessive resorption by binding to RANKL and preventing it from interacting with RANK. Thus, the relative concentration of RANKL and OPG is a major determinant of bone mass and strength (Boyce and Xing, 2008). Blocking Cx43 gap junctions and HCs resulted in an elevated RANKL/OPG ratio in differentiated mature osteocytes, suggesting a potentially catabolic environment. Indeed, the CM from Cx43 KD IDG-SW3 cells induced more TRAP-positive osteoclasts and multi-nuclei mature osteoclasts after RAW264.7 cell differentiation. Previous reports have shown that the RANKL/OPG ratio increased in MLO-Y4 cells lacking Cx43 (Bivi et al., 2012; Davis et al., 2017). Osteoclast precursors cocultured with Cx43-silenced MLO-Y4 cells or treated with CM from Cx43-silenced MLO-Y4 cells enhance osteoclast differentiation (Davis et al., 2017). Consistently, one recent study reveals that Cx43 exerts protective effects against excess osteoclastogenesis *via* passing cyclic adenosine monophosphate (cAMP) between osteoblasts (Kawatsura et al., 2022). In addition, increased osteocyte apoptosis has been found in Cx43-silenced MLO-Y4 cells compared to control, which may lead to more osteoclastogenesis (Bivi et al., 2012; Davis et al., 2017). The aforementioned findings highlight the indispensable role of Cx43 in regulating both arms of bone remodeling.

Osteocytes are highly mechanosensitive cells. Our previous *in vitro* studies have shown that FFSS opens Cx43 HCs, leading to the release of anabolic factor, PGE_2_ in osteocytes (Cherian et al., 2005; Siller-Jackson et al., 2008). Our recent *in vivo* study reveals that osteocytic Cx43 HCs play a key role in endosteal anabolic responses to mechanical stimulation (Zhao et al., 2022). Interestingly, Cx43 deletion in osteoblasts/osteocytes driven by the Bglap2 promoter, or in osteocytes driven by the 8 kb DMP1 promoter, shows an enhanced periosteal response to mechanical loading (Zhang et al., 2011; Grimston et al., 2012; Bivi et al., 2013). Mechanically stimulated osteocytes release factors that increase ALP activity and calcium deposition in osteoblasts while decreasing large-sized TRAP-positive osteoclasts (Xu et al., 2019). The outcome and cell model developed in this study will help our understanding of the underlying mechanism of important biological function of osteocytes and unveil therapeutic implications for future investigations.

## Data Availability

The data sets presented in this article are not readily available. Requests to access the data sets should be directed to jiangj@uthscsa.edu.
